# Vocabulary Is an Appropriate Measure of Premorbid Intelligence in a Sample with Heterogeneous Educational Level in Brazil

**DOI:** 10.1155/2014/875960

**Published:** 2014-04-01

**Authors:** Maira Okada de Oliveira, Ricardo Nitrini, Mônica Sanches Yassuda, Sonia Maria Dozzi Brucki

**Affiliations:** ^1^Behavioral and Cognitive Neurology Unit, Faculdade de Medicina da Universidade de São Paulo (FMUSP), 255 Avenue Dr. Enéas de Carvalho Aguiar, São Paulo 05403-900, SP, Brazil; ^2^Cognitive Disorders Reference Center (CEREDIC), Faculdade de Medicina da Universidade de São Paulo (FMUSP), 206 Rua Arruda Alvim, Cerqueira Cesar, São Paulo 05419-020, SP, Brazil; ^3^Santa Marcelina Hospital, Neurology Department, 360 Rua Santa Marcelina, Vila Carmosina, São Paulo 08270-070, SP, Brazil; ^4^School of Arts, Sciences and Humanities, Universidade de São Paulo, 1000 Avenue Arlindo Bettio, São Paulo 03828-000, SP, Brazil

## Abstract

Crystallized intelligence refers to one's knowledge base and can be measured by vocabulary tests. Fluid intelligence is related to nonverbal aspects of intelligence, depends very little on previously acquired knowledge, and can be measured by tests such as Block Design (BD) and Raven Colored Matrices (RCM). Premorbid intelligence quotient (IQ) refers to one's intellectual ability level previous to the onset of disorders like mild cognitive impairment (MCI) and Alzheimer's disease (AD) and it is important to estimate disease severity. The objective was to compare performance in tests that measure crystallized and fluid intelligence in healthy subjects and patients with amnestic MCI (aMCI) and AD. One hundred forty-four participants (aMCI (*n* = 38), AD (*n* = 45), and healthy controls (*n* = 61)) were submitted to neuropsychological tests (WAIS-III vocabulary, BD, and RCM). There were significant among groups, except for vocabulary, indicating a relative stability of crystallized intelligence in the continuum from normal to pathological cognitive decline. Vocabulary seems to be stable during the progression of the disease and useful as a measure of premorbid intelligence, that is, to estimate previous function in relation to the level of education and, as a collateral measure of cognition in people with low education.

## 1. Introduction

In the early 1940s, two types of intelligence were distinguished [1]. One type of intelligence was described to have a physiological substrate, to reach its peak in early adulthood, and to decline linearly with age. The other type was purported to be influenced by cultural experience and to remain preserved throughout most of adulthood. Years later, a bifactorial model of intelligence described the former type as fluid and the latter as crystallized intelligence [2]. This model proved to be quite influential in the field of Gerontology as longitudinal studies confirmed their differential paths of change in adulthood [3].

Crystallized intelligence is associated with an individual's knowledge base, such as knowing the vocabulary and rules of the language and historical facts. It encompasses skills required in the navigation of everyday situations and it is usually associated with the notion of “social intelligence” or “common sense” [4]. Crystallized intelligence is influenced by educational experience. Vocabulary and world knowledge are tasks frequently used to assess this type of intelligence [5–7]. Previous studies have repeatedly shown that crystallized intelligence remains stable in adulthood [3].

On the other hand, fluid intelligence relates to tasks that depend little on previously acquired knowledge and culture. It is thought to be composed of mental operations that individuals engage in when faced with a relatively new challenge, which cannot be executed automatically [4, 8]. Longitudinal studies have reported that it follows a linear trajectory of decline after the third decade of life [9, 10]. Tests such as Block Design (BD) [5] and Raven Colored Matrices (RCM) are frequently used to measure fluid intelligence as they require the ability to solve novel problems [10–13]. Traditional measures of fluid intelligence may also include perceptual speed and mental rotation [14].

Crystallized and fluid intelligence measures, such as Vocabulary and BD, respectively, have been used in neuropsychology to generate an estimate of the intelligence quotient (IQ) [15–20]. Although the IQ index has been highly questioned in its ability to predict future outcomes in children and adolescents, in the cognitive aging field, it has been used as a proxy measure for cognitive reserve, that is, one individual's ability to withstand neuropathological lesions before showing cognitive decline [21]. Knowing one's premorbid IQ, or the estimated intellectual performance level that a person had before the onset of a disease, may be useful in assessing the magnitude of cognitive decline in mild cognitive impairment (MCI) and Alzheimer's disease or other dementia subtypes. For this purpose, ideal premorbid IQ measures should be little impacted by neurocognitive disease. Using premorbid IQ measures that change as the disease progresses could lead to the underestimation of dementia severity.

Premorbid IQ has been measured by tests based on reading ability, such as the National Adult Reading Test (NART) [22] or Wide Range Achievement Test-Revised (WRAT-R) [23]. Vocabulary tests have also been extensively used [24–27]. This practice rests on the assumption that these linguistic abilities will only decline in the later stages of neurodegenerative diseases such as AD. In fact, some studies have shown that tests measuring linguistic abilities do not differ in healthy individuals, MCI, and AD [28–31]. On the other hand, Taylor [32] found that performance on the NART is clearly influenced by severity of dementia. Therefore, some authors have suggested that premorbid abilities should be estimated based on demographic variables, such as education, social class, and occupational attainment or level of engagement in cognitive activities [33].

In developing countries, the evaluation of cognition in individuals with low levels of education is a major challenge, as low cognitive performance may be due to neurological disorder or low exposure to formal education, and heterogeneous quality of schooling, as well. Therefore, in this context, assessment of premorbid IQ might be useful to estimate cognitive change [21]. To date, little is known about ideal measures of premorbid intelligence in the context of heterogeneous education background, as most studies in this field have been conducted in high-income countries.

The present study was designed to compare performance of tests that measure crystallized and fluid intelligence in healthy older adults and patients with amnestic MCI (aMCI) and AD. Our aim was to determine which tests would be stable among the diagnostic groups, so that they may be used as a measure of premorbid intelligence in a sample with heterogeneous educational levels.

## 2. Methods

### 2.1. Participants and Procedures

The sample consisted of 144 older adults who were classified into three levels of cognitive performance: normal controls (NC) (*n* = 61), patients diagnosed as having amnestic mild cognitive impairment (aMCI) (*n* = 38), or patients diagnosed as having AD (*n* = 45).

The following criteria were used for inclusion in the study: at least one year of formal or informal education, being able to read the one sentence, being 60 years of age and older, and presence of an informant. Exclusion criteria included having 15-item Geriatric Depression Scale score higher than six points and the presence of other psychiatric disorders or sensory deficits that might hinder cognitive testing [30]. Participants were not excluded from the study if they were taking antidepressants (for at least two months in stable doses) and GDS scores were six and lower.

NC were healthy volunteers, without history of neurological or psychiatric diseases, who showed no evidence of cognitive impairment and depression (lower than six) [34]. Healthy controls were included in the study if they fulfilled these criteria: absence of cognitive decline according to education-adjusted cut-off scores of the Mini-Mental State Examination (MMSE) [35] and a score lower than two on the Functional Activities Questionnaire (FAQ) [36]. They were recruited at outpatient units at the hospitals mentioned below which assisted patients who had conditions which did not affect cognition, such as internal medicine.

Patients with aMCI and AD were recruited from the Cognitive Neurology Outpatient Clinic at the University of São Paulo School of Medicine and the Cognitive Neurology Outpatient Clinic from the Santa Marcelina Hospital in São Paulo, Brazil.

Diagnostic criteria for probable AD were based on NINCDS-ADRDA [37]. All AD patients were being treated with stable doses of cholinesterase inhibitors. Patients were excluded from the study if they had moderate or severe dementia, dementia caused by other etiologies, and a Cornell Scale for Depression in Dementia score higher than seven [38].

Diagnostic criteria for aMCI were based on the international consensus by Winblad et al. [39] aMCI patients had scores lower than five in the FAQ and cognitive impairment in at least one cognitive test including an episodic memory measure. The aMCI group included patients with impairment exclusively in episodic memory (single domain aMCI) and in episodic memory and other cognitive domains (multiple domain aMCI). Cognitive impairment was identified when performance in cognitive tests (adjusted by education and age) was below 1.5 standard deviations (SD).

### 2.2. Instruments

All patients and NC participants were evaluated by neuropsychologists and neurologists of the services mentioned above. They completed a comprehensive neuropsychological battery and their informants completed functional evaluation questionnaires, as described below. Laboratory and neuroimaging exams were carried according to the recommended consensus criteria of the Brazilian Academy of Neurology [40]. Clinical diagnosis was completed by trained neurologists after the review of all the available clinical information.

The neuropsychological assessment included the following tests: Dementia Rating Scale [41, 42], Mini-Mental State Examination [35, 43], Rey Auditory Verbal Learning Test (RAVLT) [44], 10 black and white pictures of the Brief Cognitive Screening Battery [45–47], Block Design (BD) and Vocabulary subtests from the Wechsler Adult Intelligence Scale (WAIS-III) [6, 48], and Raven's Colored Matrices (RCM) [49, 50].

In the present analysis, BD and RCM were used as measures of fluid intelligence and Vocabulary was used as a measure of crystalized intelligence. Estimated IQ scores were calculated by summing the standardized scores of BD and Vocabulary, according to usual procedures [17].

This study was approved by the Ethics Committee of the Hospital das Clinicas from the University of São Paulo School of Medicine and Hospital Santa Marcelina under the Protocol no. 0632/09. All subjects who agreed to participate, or the caregiver of the dementia patients, signed a written informed consent.

### 2.3. Statistical Analysis

Normal distribution was evaluated with Kolmogorov-Smirnov test. As most cognitive variables did not follow normal distribution, diagnostic groups were compared with the Kruskal-Wallis test and multiple comparisons test. For categorical variables such as sex, the chi-square test was used. Spearman's correlation analyses were calculated to investigate the associations among age, education, MMSE, Vocabulary, BD, and RCM.

Two regression analyses were computed with clinical diagnosis as the dependent variable (NC × MCI and NC × AD) and age, education, sex, Vocabulary, BD, and RCM as independent variables. All statistical analyses were carried out using the* Statistical Package for the Social Sciences* (SPSS) program, version 15.0. The level of significance accepted was *P* < 0.05.

## 3. Results 

There were no significant differences among groups regarding gender distribution and education. As to age, the AD group was older than NC and aMCI (Table 1).

There were significant differences between the NC and the aMCI and the AD groups for the MMSE, BD, RCM, and estimated IQ. No differences were observed among the three groups for Vocabulary, indicating stability of this variable across diagnostic groups (Table 2).

Spearman correlations (Table 3) indicated that age was not associated with MMSE scores. Education was associated with MMSE scores in the three diagnostic groups. Vocabulary, BD, and RCM were associated with MMSE scores among NC and aMCI participants, but not among AD patients.

Regression analyses, with clinical diagnosis as the dependent variable, suggested that the scores for RCM were associated with aMCI diagnosis (Table 4) and BD and age were associated with AD diagnosis. Scores for Vocabulary are not significantly associated with MCI or AD diagnosis.

## 4. Discussion 

The goal of the present study was to compare the performance of healthy controls, aMCI, and AD patients with heterogeneous levels of education on measures of crystallized and fluid intelligence so that an appropriate measure of premorbid intelligence could be identified. Vocabulary scores were statistically equivalent across the three diagnostic groups, suggesting that it would be an appropriate measure of premorbid IQ in a sample with heterogeneous educational background. Regression analyses confirmed these results, as Vocabulary scores were not associated with diagnosis of aMCI or AD.

In our results, fluid intelligence declined with increasing cognitive impairment, whereas crystallized intelligence was well preserved. Other studies have reported similar results [13, 31, 51].

Having both premorbid and current measures of intelligence may be useful in determining the severity of the disease affecting the patient, because the examiner is able to estimate the patient's premorbid abilities (using a Vocabulary test) and how much the disease has impaired his or her current performance (using RCM and BD). In fact, cognitive scores may need to be interpreted according to premorbid IQ levels, as suggested by recent studies in Portugal [51]. These measures may also be useful in a rehabilitation program as Vocabulary scores may indicate visible intervention strategies.

In patients with limited education, it may be difficult to interpret fluid intelligence measures (current intelligence), as low scores might be due to lack of education or due to neurodegenerative processes affecting regions used to perform the tests. By using the Vocabulary subtest, the examiner may have some knowledge of the premorbid intellectual resources of this patient. Clinicians currently use demographic information (education and occupation) to estimate previous functioning; however, some patients have greater cognitive reserve through lifelong habits (i.e., reading, writing and high demand activities) and often surpass patients who may have more years of formal education. Questionnaires may be used to estimate reserve associated with intellectual engagement, yet, such questionnaires need to be validated for specific populations, whereas WAIS Vocabulary subtest is validated and normed for most nations, enabling cross-cultural comparisons.

The limitations of this study were that the examiner was not blinded to diagnosis and there were age differences between groups; however, age did not correlate with any cognitive results. In conclusion, Vocabulary seems to be a measure of premorbid intelligence and it may be useful to estimate previous function in relation to educational level and the magnitude of cognitive change.

## Figures and Tables

**Table 1:** Demographic characteristics of the sample divided into diagnostic groups.

	NC (*n* = 61)				aMCI (*n* = 38)				AD (*n* = 45)				*P *
	Mean	SD	Min	Max	Mean	SD	Min	Max	Mean	SD	Min	Max	
Gender (F/M)	35/26				24/14				26/19				0.83
Age	70.66	(6.55)	60	91	72.32	(7.94)	61	89	75.80	(4.81)	65	86	<0.01^a^
Education	8.72	(5.44)	1	21	7.03	(4.87)	0	16	6.96	(4.57)	1	19	0.13

Note. *P* value for gender comparisons refers to the chi-square test, and, for age and education, it refers to the Kruskal-Wallis test; ^a^NC differ from AD; aMCI differs from AD. F: female; M: male; SD: standard deviation; NC: normal controls; aMCI: amnestic mild cognitive impairment; AD: Alzheimer's disease; Min: minimum value; Max: maximum value.

**Table 2:** Cognitive performance for NC, MCI, and AD.

Tests	NC	aMCI	AD	P*
	Mean (SD)	Mean (SD)	Mean (SD)	
MMSE	28.36 (1.48)	25.79 (2.74)	23.96 (2.90)	<0.01^a^
Vocabulary	26.46 (9.81)	22.81 (9.38)	23.20 (9.16)	0.146
BD	25.43 (8.93)	18.39 (8.53)	14.18 (6.53)	<0.01^b^
RCM	25.84 (6.23)	20.47 (6.31)	17.76 (5.99)	<0.01^b^
Estimated IQ	98.77 (10.41)	91.08 (10.13)	87.95 (8.92)	<0.01^b^

Note. *P* value refers to Kruskal-Wallis test; SD: standard deviation; MCI: mild cognitive impairment; AD: Alzheimer's disease; MMSE: Mini-Mental State Exam; IQ: intelligence quotient; BD: Block Design; RCM: Raven Colored Matrices.

^a^All groups differ.

^b^NC differ from AD and aMCI; MCI is equal to AD.

**Table 3:** Correlations between the MMSE and age, education, and intelligence variables.

Group	Screening	Age	Education	Vocabulary	BD	RCM
NC	MMSE	0.077	0.467**	0.574**	0.343*	0.448**
aMCI	MMSE	−0.107	0.502**	0.431*	0.616**	0.596**
AD	MMSE	0.165	0.327*	0.199	0.174	0.80

Note. *P < 0.05; **P < 0.01; MMSE: Mini-Mental State Exam; BD: Block Design; RCM: Raven Colored Matrices; aMCI: amnestic mild cognitive impairment; AD: Alzheimer's disease.

**Table 4:** Multivariate logistic regression analyses with clinical diagnosis as the dependent variables and age, education, sex, Vocabulary, BD, and RCM as independent variables.

NC × aMCI	Adjusted *R* ^2^	0.267
	*B* (*P* value)	RCM = −0.164 (*P* < 0.001)

NC × AD	Adjusted *R* ^2^	0.499

	*B* (*P* value)	Age = 0.10 (*P* = 0.030)
	*B* (*P* value)	BD = −0.195 (*P* < 0.001)

Note. MMSE: Mini-Mental State Exam; BD: Block Design; RCM: Raven Colored Matrices; aMCI: amnestic mild cognitive impairment; AD: Alzheimer's disease.
